# Physiologically Based Pharmacokinetic Model for Prediction of Immunoglobulins Exposure in Pregnant Women

**DOI:** 10.3390/antib14040099

**Published:** 2025-11-19

**Authors:** Million A. Tegenge

**Affiliations:** Office of Biostatistics and Pharmacovigilance (OBPV), Center for Biologics Evaluation and Research (CBER), Food and Drug Administration (FDA), Silver Spring, MD 20993-0002, USA; million.tegenge@fda.hhs.gov; Tel.: +1-240-402-8802

**Keywords:** dosing, model-informed drug development (MIDD), pregnancy, PBPK, antibodies

## Abstract

**Background**: Physiologically based pharmacokinetic (PBPK) modeling is applied to address clinical pharmacology issues including dose selection and exposure assessments for special populations (e.g., pediatrics, and renally or hepatically impaired patients). The objective of this study was to evaluate the predictive performance of a PBPK model for dosing assessment of intravenous immunoglobulin (IVIG) and anti-D immunoglobulin (anti-D Ig) products in pregnant women. **Methods**: A minimal PBPK (mPBPK) model that incorporates pregnancy-specific physiological parameters and allometric scaling approaches was developed and evaluated for predicting the exposure of IVIG and anti-D Ig in pregnant women. The concentration versus time data were obtained from the published literature. **Results:** The IVIG (n = 22) and anti-D Ig (n = 29) concentrations were predicted using the mPBPK model with an average fold error of 1.17 and 1.22, respectively. A total of 100% and 95% of IVIG concentrations were predicted within the 0.5–2-fold and 0.5–1.5-fold prediction error ranges, respectively. For anti-D Ig, predictions fell within the 0.5–2-fold and 0.5–1.5-fold ranges for 93% and 76% concentrations, respectively. A mPBPK model-based simulation following administration of 0.5 g/kg IVIG in 100 virtual nonpregnant and pregnant subjects revealed that the maximum plasma concentration (Cmax) was 15% lower and trough concentration (Ctrough) was 8% lower during the third trimester of pregnancy compared to nonpregnant subjects. In contrast, with flat dosing, Cmax and Ctrough were 32% and 26% lower in pregnant subjects, respectively. Overall, the model demonstrated reasonable predictive performance, and bodyweight-based dosing regimen is an acceptable approach that results in minimal change in exposure of IVIG in pregnant women.

## 1. Introduction

Intravenous immunoglobulin (IVIG) products are commonly used for treating immunodeficiency diseases such as primary humoral immunodeficiency (PID) [1,2,3]. There are several off-label uses of IVIG products to treat non-obstetrical- and obstetrical-related conditions in pregnant women [4]. In contrast, Rh0 (D) immunoglobulin intravenous or intramuscular products (i.e., anti-D Ig) are specifically indicated for pregnancy and obstetric conditions in non-sensitized, Rh0 (D)-negative women with a Rh-incompatible pregnancies [5,6].

The pharmacokinetics (PK) of IVIG have been well characterized, and PK information has been incorporated into FDA approval decisions for IVIG products used to treat of PID in adults and, in some cases, for children (≥2 years of age) [2,3,7,8,9]. However, such PK data are not available in pregnant women for FDA-licensed IVIG products, creating a significant knowledge gap.

Determining appropriate dosing for therapeutic antibodies in pregnant women presents a significant clinical challenge due to the exclusion of this population from most clinical trials. Currently, pregnant women receiving IVIG products typically receive empirical doses based on standard adult regimens, despite substantial physiological changes during pregnancy that may alter drug disposition. During pregnancy, multiple factors can influence antibody disposition, including increased plasma volume, altered bodyweight, changes in protein binding, and modified clearance mechanisms. During pregnancy, fetal immunoglobulin G (IgG) levels increase dramatically (5- to 8-fold) after the third trimester compared to levels at early stages [10]. This increase is clinically relevant, as similar IgG transfer to the fetus has been reported following administration of high doses of exogenous IVIG in pregnant women after 32 weeks of pregnancy [11]. The mechanism underlying this increase in fetal IgG levels after the third trimester involves active transport mediated by the neonatal Fc receptor (FcRn). Importantly, the FcRn is expressed on the syncytiotrophoblast and endosomal membranes at increasing levels after the third trimester but is barely detectable before the 14th week of gestation [12,13,14].

Model-informed drug development (MIDD) approaches are intended to facilitate the development and application of exposure-based, biological, and statistical models derived from preclinical and clinical data sources. Among such MIDD approaches that are applied to address drug development issues in clinical pharmacology is physiologically based pharmacokinetic (PBPK) modeling [15,16,17]. PBPK models offer unique advantages to integrate diverse types of data to make predictions of PK and exposure in pregnant women. PBPK modeling has been applied to predict exposure and PK of small molecule drugs in pregnant women [18,19,20,21,22,23]; however, there is no published PBPK model for prediction of exposure of immunoglobulins product in pregnant women. Several factors may limit the ability to develop a complex mechanistic PBPK model. These include the physiological changes during pregnancy, the complexity of the PK characteristics of antibodies, and the lack of human PK studies in pregnant women.

The objective of this study was to develop and evaluate an empirical minimal PBPK (mPBPK) model for exposure assessment of IVIG and anti-D Ig during pregnancy. By incorporating pregnancy-specific physiological parameters and allometric scaling approaches, the model provides a scientific framework for evaluating the appropriateness of bodyweight-based dosing strategies in the absence of extensive clinical trial data.

## 2. Methods

### 2.1. Data

The concentration versus time data from published studies were extracted using WebPlotDigitizer (version 4.2, https://apps.automeris.io/wpd/, accessed on 10 March 2024). The PK data for developing the mPBPK were retrieved from two studies [24,25]. In these studies, IVIG was administered at doses ranging from 300 to 600 mg/kg in adult subjects [24], and anti-D Ig was administered at a dose of 300 µg in women at the 28th week of pregnancy via either by the intramuscular or intravenous route [25]. The predictive performance of the mPBPK model was evaluated using separate PK data obtained from five published studies [11,25,26,27,28] that are summarized in Table S1.

### 2.2. Nonpregnancy PBPK Model Development

The PBPK model was developed using a previously published minimal PBPK (mPBPK) modeling framework for monoclonal antibodies [29] and details including equations and schematics are provided in the Supplementary Information (Table S2 and Figure S1). In the mPBPK model, leaky tissues include liver, kidney, heart, and other highly vascularized organs, while tight tissues include muscle, skin, adipose tissue, and brain [29]. The physiological parameters for calibrating the typical adult mPBPK model were retrieved from a published study [29] and are summarized in Table 1. The antibody-specific parameters such as clearance and vascular reflection coefficients for both tight tissues (σ_1_) and leaky tissues (σ_2_) were estimated using the training data (Table 1 and Table S3).

### 2.3. Pregnancy PBPK Model for Predicting Exposure of Immunoglobulins

The basic adult mPBPK model and parameter estimates for IVIG were scaled based on the bodyweight and physiological parameters of pregnancy as shown in Table 1. The pregnancy-related physiological compartments such as placenta and fetus were incorporated in the leaky compartment of the mPBPK model [19]. In the absence of mechanistic and clinical data, the lymphatic reflection coefficient and vascular reflection coefficients for both leaky and tight tissues were assumed to remain the same for nonpregnancy and pregnancy scenarios. The baseline IgG levels for nonpregnancy, and pregnant women at first, second, and third trimesters were compiled from published studies [10,31] and incorporated into the mPBPK model for prediction of PK of IgG in pregnant women. For simulation of anti-D Ig following intramuscular administration, the absorption rate constant was obtained from a published study [32]. Drug-specific parameters such as σ_1_, σ_2,_ and clearance were estimated by fitting concentration vs. time data following intravenous administration of anti-D Ig in women in their third trimester of pregnancy [25]. Pregnancy-related changes in FcRn expression may influence IgG kinetics; however, due to the lack of quantitative data on FcRn expression across different stages of pregnancy, this effect was assumed to be grossly captured through the clearance term in the model (Figure S1).

### 2.4. Simulation of IVIG PK in Nonpregnant and Pregnant Women

A mPBPK model-based simulation was conducted to quantify the exposure difference between nonpregnant and pregnant women following IVIG administration using bodyweight-based dosing of 0.5 g/kg. The bodyweight of 100 virtual nonpregnant women was obtained from the CDC database [33] and 23% increase in bodyweight was incorporated for the description of bodyweight during the third trimester of pregnancy [30,34]. The PK profiles of 100 nonpregnant and pregnant women were simulated using the mPBPK model. The exposure parameters, Cmax, and Ctrough were compared between nonpregnant and pregnant women.

### 2.5. Software and Statistical Evaluations

The mPBPK model was developed, and concentration vs. time profiles were simulated using the nonlinear mixed-effect modeling software (NONMEM VII 3.0; ICON Development Solutions, Hanover, MD, USA).

Average fold error (AFE), which is the log transformed ratio of the predicted and observed concentrations, was calculated. For AFE, a value of 1.0 indicates no prediction error and AFE was calculated as follows:

(1) AFE=101/N∑log(Pred,iObs,i)

where *N* is the total number of observations, and Pred,i and Obs,i are predicted and observed IgG concentrations, Cmax or AUC for each study, respectively.

Percent prediction error between the observed and predicted values was calculated according to the following equation:

(2) $$\%\mathrm{Prediction}\;\mathrm{error}=100\times \frac{\left(\mathrm{Predicted}-\mathrm{Observed}\right)}{\mathrm{Observed}}$$

## 3. Results

### 3.1. Development of PBPK Model

**Figure 1:**
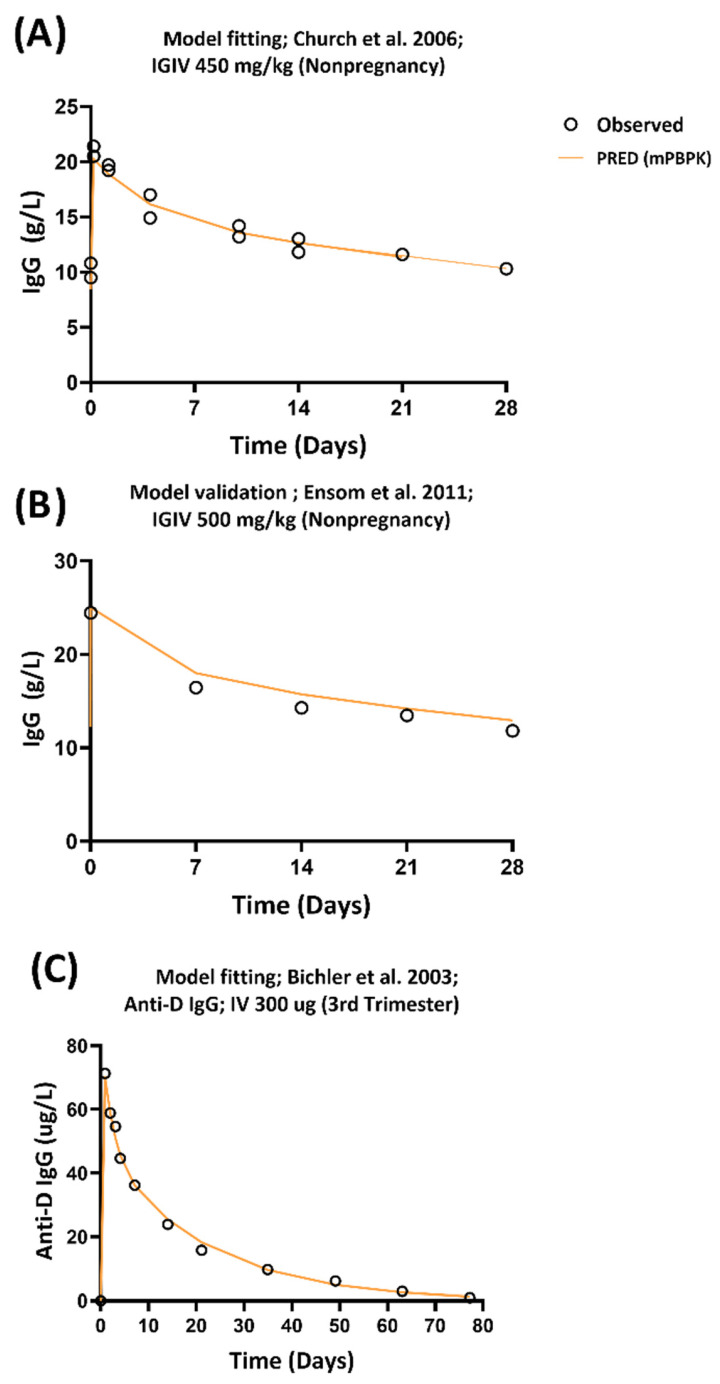
The observed and model predicted IVIG and anti-D Ig concentrations in nonpregnant and pregnant subjects. (**A**) IVIG concentration profile obtained from Church et al. 2006 [24] and mPBPK fitted curve (orange) and (**B**) external validation of mPBPK model with PK data from nonpregnant subjects obtained from Ensom et al. 2011 [26]. (**C**) Anti-D Ig concentration following intravenous administration obtained from Bichler et al. 2003 [25] and mPBPK fitted curve (orange). List of PK studies of IVIG and anti-D Ig is shown in Table S1.

The mPBPK model was calibrated to predict the observed plasma concentrations from the training PK data for IVIG [24]. For the mPBPK, a typical adult subject’s PK data was simulated with a dose of 450 mg/kg of IVIG (i.e., the average dose administered in the observed PK data). The input model parameters (vascular reflection coefficients for tissues and plasma clearance) were estimated with a good precision. The mPBPK model captured the observed plasma concentration versus time profile of IVIG for the training data (Figure 1A).

The predictive performance of the mPBPK model was further evaluated with a separate external PK data obtained from nonpregnant women [26]. It should be noted that for the validation of the external data, the input parameters derived using the training data were directly used without further estimation of parameters. The predicted plasma concentration versus time profile following simulation of IVIG PK in a typical nonpregnant subject matched the observed profile of IgG for the validation data (Figure 1B).

The mPBPK model for describing anti-D Ig plasma concentration was developed by employing PK data from women at the 28th week of pregnancy following intravenous administration of 300 µg of anti- D Ig [25]. The input model parameters were estimated with a good precision (Table S3) and the mPBPK model captured the observed plasma concentration versus time profile of anti-D Ig following intravenous administration (Figure 1C).

### 3.2. Evaluation of mPBPK Models in Pregnant Women

**Figure 2:**
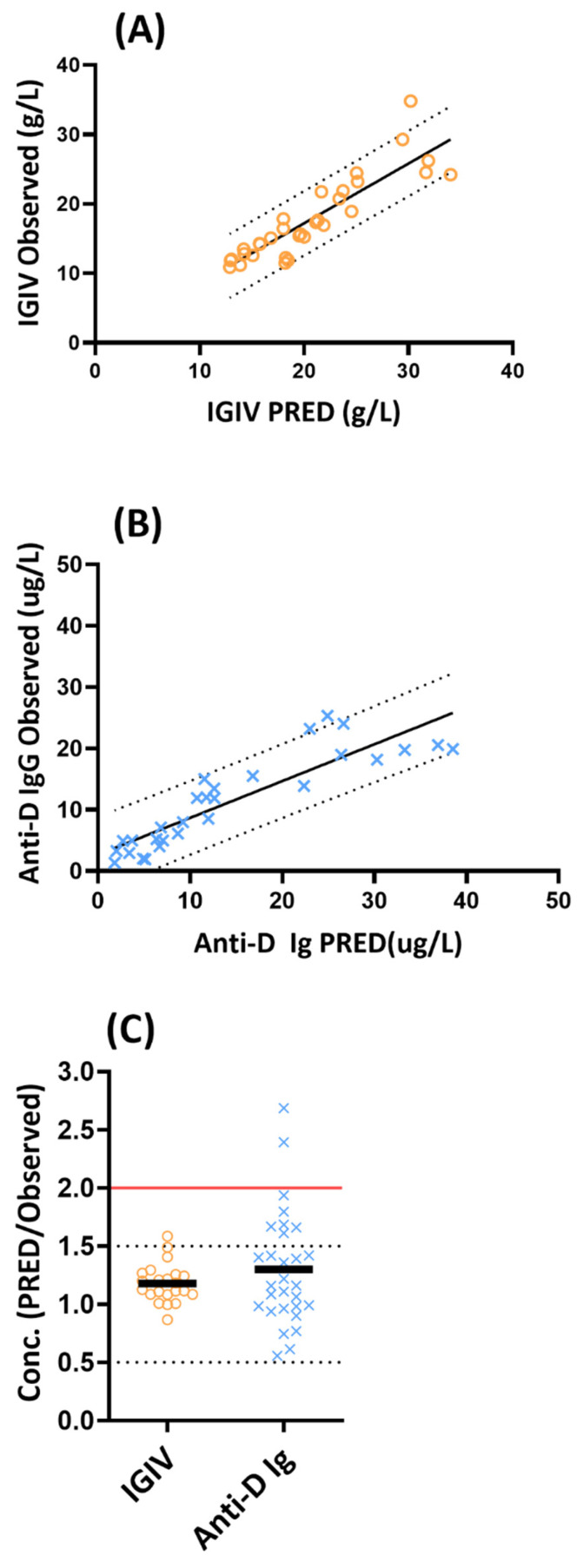
Pregnancy mPBPK model evaluation. (**A**) Plot of the predicted IVIG versus observed concentrations, (**B**) plot of the predicted anti-D Ig versus observed concentrations, and (**C**) the ratio of concentration predicted (PRED) to observed was plotted for IVIG and anti-D Ig. The solid lines in (**A**,**B**) represent linear regression and dotted lines mark the 90% prediction interval. The dotted horizontal line in (**C**) marks the 0.5- to 1.5-fold range and the dark solid line shows the mean value for the fold error. The red solid line indicates the 2-fold prediction range.

The predicted versus observed plasma concentration profiles of IVIG and anti-D Ig in pregnant women were displayed in Figure 2A,B. The overall ratio of mPBPK model predicted to observed concentrations (n = 22 for IVIG and n = 29 for anti-D Ig) was shown in Figure 2C. The predictive performance of the mPBPK model for IVIG and anti-D Ig are described below.

**IVIG:** The mPBPK model was validated by employing PK data obtained from two studies that collected IgG concentrations following IVIG administration in pregnant women in their first, second, and third trimester (Table S2). As shown visually in Figure 2A, the mPBPK model reasonably predicted the overall concentration profiles of IVIG in pregnant women. From the mPBPK model (n = 22), 100% and 95% observations were within 0.5–2.0 and 0.5–1.5-fold prediction error, respectively (Figure 2C). The accuracy of the prediction was evaluated by AFE, where a value of 1.0 indicates no prediction error. The AFE for mPBPK model was 1.17, indicating a good overall prediction with a slight bias toward over prediction of IVIG concentrations.

**Anti-D Ig:** The PK data for anti-D Ig was obtained from three studies that characterized PK following intramuscular administration of 100–300 μg of anti-D Ig in pregnant women in their third trimester (Table S1). As shown visually in Figure 2B, the mPBPK model reasonably predicted the overall concentration profiles of anti-D Ig in pregnant women. From the mPBPK model (n = 29), 93% and 76% observations were within 0.5–2.0 and 0.5–1.5-fold prediction error, respectively (Figure 2C). Two concentrations (7%) were predicted with a 2.4- and 2.7-fold error from the observed concentrations (Figure 2C). The AFE for the mPBPK model is 1.22, indicating a good overall prediction with a slight bias toward over prediction of anti-D Ig concentrations.

Overall, the mPBPK model reasonably predicted IVIG and anti-D Ig concentrations (n = 51) with 96% of the predicted concentrations within the 0.5–2-fold range.

### 3.3. Quantification of Exposure Difference in Nonpregnant Versus Pregnant Women Following IVIG Administration

**Figure 3:**
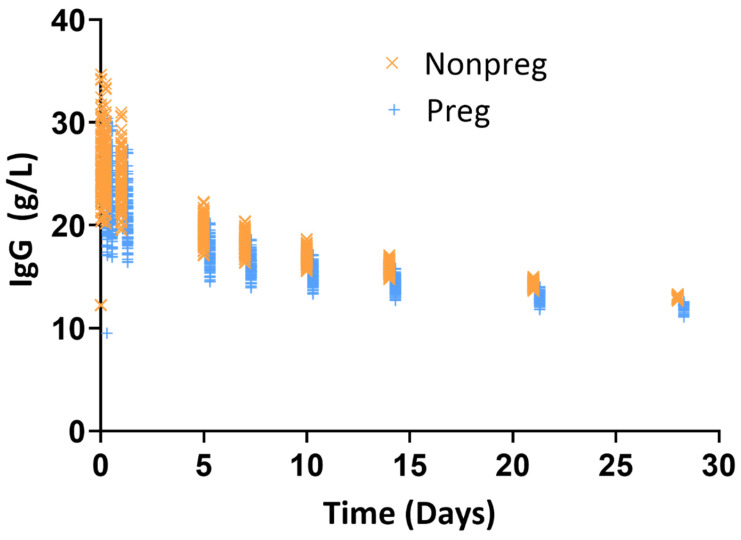
Model simulation of IVIG the concentration versus time profile in 100 virtual nonpregnant and pregnant subjects following 0.5 g/kg bodyweight-based administration. Orange and blue symbols represent individual subject-level concentrations for nonpregnant and pregnant (third trimester) scenarios, respectively. The overlapping concentration profiles demonstrate that bodyweight-based dosing effectively mitigates exposure differences between pregnancy states, with minimal separation observed, particularly during early timepoints.

Total bodyweight is used for standard dosing of FDA-approved IVIG products. Here, mPBPK model-based Monte Carlo simulation was performed to quantify the exposure difference between nonpregnant and pregnant women using bodyweight-based dosing of 0.5 g/kg. For simulation, the third trimester was selected, since previous PK study has showed no major exposure difference between nonpregnant and pregnant women in their first and second trimester [26] and major change in bodyweight and other physiological changes relevant for disposition of IgG occur in the third trimester [30]. The result of the simulation was summarized in Table 2 and Figure 3. As shown in Figure 3, the simulated concentrations were overlapping between nonpregnant and pregnant women. Because of the bodyweight difference, the administered total dose was 24% higher for pregnant versus nonpregnant women (Table 2). The Cmax and Ctrough was 15% and 8% lower, respectively, in pregnant women as compared to nonpregnant women. When the Cmax and Ctrough were adjusted for the difference in dosing (i.e., assuming equal total dose based on flat dosing), the Cmax and Ctrough were 32% and 26% lower in pregnant women as compared to nonpregnant women (Table 2). These results demonstrated that bodyweight-based dosing, in part, compensate for the expected higher drop in PK/exposure of IVIG in pregnant women as compared to nonpregnant women.

## 4. Discussion

Pregnant women are often excluded from clinical trials and hence, at the time of a drug’s initial marketing, there are typically no human data on the appropriate dosage and frequency of administration during pregnancy. In the absence of such data, the usual adult dose is typically prescribed for pregnant women [35]. This practice appears to be the case for IVIG products, which are commonly used off-label to treat non-obstetrical and obstetrical conditions in pregnant women using empirical dosing based on regimens for typical adult subjects [4]. A PBPK modeling-based extrapolation of PK information has been previously evaluated for small molecule drugs for exposure and dosing assessments [18,19,20,21,22,23]. Building on this foundation, this study aimed to develop and evaluate the suitability of mPBPK as a potential modeling and simulation (M&S) tool for extrapolating exposure of IVIG and anti-D Ig in pregnant women.

The proposed mPBPK model was initially developed and validated in typical nonpregnant subjects. The modeling framework of this mPBPK is comparable to previously published models for monoclonal antibodies in nonpregnancy scenarios [29,36]. For validation of mPBPK in pregnant populations, the PK data for IVIG were obtained from studies that collected concentration versus time profiles from pregnant women in their first and second trimesters [26] and limited concentrations were available from pregnant women in their third trimester [11]. In contrast, the PK of anti-D Ig products have been more commonly studied in pregnant women [5,6,25], providing PK data to validate the predictive performance of the mPBPK model in pregnant women in their third trimester. The current study results indicate that the mPBPK model provided acceptable prediction of IVIG and anti-D Ig concentrations (n = 51) with 96% of the predicted concentrations within the 0.5–2-fold range for pregnant women.

In the absence of adequate clinical PK data in pregnant women for IVIG products, pregnancy-related physiological compartments such as the placenta and fetus were incorporated into leaky compartments of the mPBPK model. A similar approach was applied for characterization of PK and dosing assessment for sertraline [19]. For therapeutic proteins, relevant physiological parameters (e.g., tissue volume, lymph volume, and lymphatic flow) and drug-related parameters (e.g., clearance) are not readily available in pregnant women. In the absence of quantitative data, these parameters were fixed based on published studies, adjusted based on bodyweight, or allometrically scaled (Table 1) for pregnant women as previously applied for interspecies scaling [37] and pediatric clearance prediction [38]. Considering these empirical assumptions and the simplicity of the mPBPK model structure, the proposed M&S is a pragmatic approach for prediction of exposure of IVIG and anti-D Ig in pregnant women. A mPBPK model is preferable over a whole-body PBPK model due to its simplicity for extrapolation, reduced parameter requirements, and ability to overcome challenges related to limited clinical data availability and parameter identifiability in pregnant populations. This empirical mPBPK approach aligns with ICH M15 principles [39] of fit-for-purpose modeling, where model complexity should match available data, prior knowledge, and intended application. The successful external validation using independent datasets supports the applicability of the model for exposure prediction and dosing determination. It should be noted that previous studies showed that mPBPK models are as robust as the more complex whole-body PBPK models for prediction of exposure of small molecule drugs and therapeutic proteins [29,36,38,40,41,42,43], suggesting that model elaboration may not add value in the absence of adequate clinical studies to quantify relevant parameters.

One practical application of the PBPK model is to evaluate if pregnancy-related physiological changes significantly change drug exposure. In this study, a mPBPK model-based simulation following administration of 0.5 g/kg IVIG in 100 virtual nonpregnant and pregnant subjects revealed that the Cmax of IVIG was lower by 15% and Ctrough was lower by 8% during the third trimester of pregnancy (Figure 3 and Table 2). However, if one assumes a flat dose (i.e., if an equal total dose was given to both nonpregnant and pregnant women), the Cmax was lower by 32% and Ctrough lower by 26% in pregnant women. The small change in exposure using bodyweight-based dosing indicates that incorporating bodyweight into dosing partially compensates for pregnancy-related PK changes. A similar observation was previously noted in pregnant women receiving IVIG treatment [26]. The 23% increase in bodyweight during the third trimester, combined with expanded plasma volume (41% increase) and other pregnancy-related physiological changes, would typically result in lower drug concentrations. However, the proportional increase in total dose through bodyweight-based dosing counteracts these dilutional effects, maintaining clinically relevant exposure levels.

The current study is limited by the availability of PK data used for model validation. Although data from five published studies were compiled for model validation of IVIG and anti-D Ig, these data are still considered limited to derive PK parameters. In addition, the available sparse clinical PK data have high variability and did not allow us to develop a detailed mechanistic model. The observed clinical PK variability may be due to factors such as age range, dose range, and biochemical assay. Clinical PK data in pregnant women across a wide gestational range of pregnancy are needed to further validate and apply the mPBPK model for dosing recommendation for immunoglobulins. Future study may also focus on developing detailed mechanistic models (e.g., including quantitative change in expression of FcRn across a different stage of pregnancy) to further improve model prediction.

## 5. Conclusions

The mPBPK model demonstrated acceptable predictive performance for both IVIG and anti-D Ig products in pregnant women, with 96% of predicted concentrations falling within the 0.5–2-fold range of observed values. The study demonstrated that bodyweight-based dosing is a reasonable approach for IVIG administration in pregnant women. During the third trimester, this dosing strategy resulted in only modest reductions in exposure compared to nonpregnant women (15% lower Cmax and 8% lower Ctrough). This mPBPK model provides a pragmatic modeling and simulation tool that can inform dosing decisions though additional clinical data across broader gestational ranges that would further strengthen its utility.

## Figures and Tables

**Table 1:** Parameters used for development and evaluation of mPBPK model for IVIG in adult subjects and pregnant women.

	Parameters for Typical 70 kg Adult [Reference]	Parameters for Pregnant Women
**Plasma volume (L)**	2.6 [29]	2.78–3.67 **
**Leaky tissue volume (L)**	4.37 [29]	4.37 * (BW/70)
**Tight tissue volume (L)**	8.11 [29]	8.11 *(BW/70)
**Lymph volume (L)**	5.2 [29]	5.2 * (BW/70)
**Total lymph flow (L/day)**	2.9 [29]	2.9 * (BW/70)
**Lymphatic capillary reflection coefficient**	0.2 [29]	0.2
**Vascular reflection coefficient**	σ_1_ * = 0.97 (0.94–0.99) σ_2_ * = 0.94 (0.93–0.95)	σ_1_ = 0.97 σ_2_ = 0.94
**Clearance (L/day)**	0.045 (0.034–0.055)	0.045 * (BW/70)^0.75^

* σ_1_ and σ_2_ indicate reflection coefficient of tight and leaky tissues, respectively. The values for σ_1_, σ_2_, and clearance were estimated, and other parameters were fixed or adjusted using bodyweight term. The values in parenthesis are 95% confidence intervals estimated with % coefficient of variation (%CV) < 10% using PK data of IVIG (nonpregnant subject). ** Plasma volume was increased by 7% at first trimester (2.78 L), 27% at second trimester (3.30 L), and 41% at third trimester (3.67 L) [30]. BW: bodyweight. Parameters used for development and evaluation of mPBPK model for Anti-D IgG in pregnant women are shown in Table S3.

**Table 2:** Minimal PBPK model-based simulation of IVIG for exposure assessments.

	Nonpregnant	Pregnant (Third Trimester)	% Mean Change
**Dose (g/kg)**	0.5	0.5	0
**Total Dose (g)**	37 ± 9	46 ± 11	24
**Cmax (g/L)**	26 ± 3	22 ± 3	−15
Cmax_adjusted (g/L) *	25	16	−32
**Ctrough (g/L)**	13 ± 0.1	12 ± 0.3	−8
Ctrough_adjusted (g/L) *	12	9	−26

* Adjusted Cmax and Ctrough were calculated based on normalized total dose for a typical 70 kg adult subject (e.g., Cmax_adjusted = Cmax × total dose for 70 kg/total dose).

## Data Availability

The original contributions presented in this study are included in the article. Further inquiries can be directed to the corresponding author(s).

## Supplementary Materials

The following supporting information can be downloaded at https://www.mdpi.com/article/10.3390/antib14040099/s1, Figure S1: Schematic representation of the minimal PBPK (mPBPK) model framework, including model inputs and outputs used for characterizing the pharmacokinetics of IGIV and anti-D Ig in nonpregnant and pregnant women. The mPBPK model categorizes tissues into leaky compartments (liver, kidney, heart, and other highly vascularized organs) and tight compartments (muscle, skin, adipose tissue, and brain) based on vascular permeability characteristics. For pregnancy applications, placenta and fetus were incorporated into the leaky tissue compartment to account for pregnancy-related physiological changes; Table S1: List of PK studies of IGIV and anti-D Ig performed in pregnant women [11,25,26,27,28,34]; Table S2: Equations used for development of minimal PBPK Model; Table S3: Parameters used for development and evaluation of mPBPK model for Anti-D IgG in pregnant women [25].
