# Supplementary material for: Physiologically Based Pharmacokinetic Model for Prediction of Immunoglobulins Exposure in Pregnant Women
Source: Antibodies (Basel). 2025 Nov 19;14(4):99. doi: 10.3390/antib14040099 (PMC12642000; doi:10.3390/antib14040099)

**Table S1:** List of PK studies of IGIV and anti-D Ig performed in pregnant women

| Autor/Reference | Number of subjects | Gestational age (Wks) at first dosing | Body weight (Kg) | Route & Dose | PK sampling | Product name |
|---|---|---|---|---|---|---|
| Ensom et al. 2011[26] | 12 | First trimester | 60.3-87 | IV; 0.5-1 g/kg | 4 post-dose samples | Gamimune or Gamunex |
| Ensom et al. 2011[26] | 10 | Second trimester | 64.2- 77.2 | IV;0.5-1 g/kg | 4 post-dose samples | Gamimune or Gamunex |
| Sidiropoulos et al.1986[11] | 11 | Second trimester | Estimated* | IV; 1x12 gm and 5x24 gm | One sample | Sandoglobulin |
| Sidiropoulos et al.1986[11] | 16 | Third trimester | Estimated* | IV; 1x12 gm & 5x24 gm | One sample | Sandoglobulin |
| Bichler et al. 2003[25] | 14 | Third trimester | 71.2 | IV & IM; 300 ug | Extensive | Anti-D IgG (Rhophylac) |
| MacKenzie et al. 2006[28] | 43 | Third trimester | 68.8-74.2 | IM: 100 ug | Extensive | Anti-D IgG |
| Tiblad et al. 2012[27] | 16 | Third trimester | 80.1 | IM; 250 ug | Extensive | Anti-D IgG (Rhesonativ) |

*Estimated using body weight equations based on the gestational weeks of pregnancy (Abduljalil et al. 2012)[34]

**Table S2:** Equations used for development of minimal PBPK Model

| Equation | Initial condition |
|---|---|
| $\frac{dCp}{dt}$=[Clymph · L – Cp · L1 · (1 –σ1) – Cp · L2 · (1 –σ2) – Cp · CLp]/Vp | Cp(0)= Dose/Vp |

| | |
|---|---|
| $\frac{dCtight}{dt}$=[Cp · L1 · (1 −σ1) − Ctight · L1 · (1 −σL)]/Vtight | Ctight(0)=0 |
| $\frac{dCleaky}{dt}$=[Cp · L2 · (1 −σ2) − Cleaky · L2 · (1 −σL)]/Vleaky | Cleaky(0)=0 |
| $\frac{dClymph}{dt}$=[ Ctight · L1 · (1 −σL) + Cleaky · L2 · (1 −σL) − Clymph · L]/Vlymph | Clymph(0)=0 |

;where $C_p$ is IgG concentration in $V_p$ (plasma volume), $C_{tight}$ and $C_{leaky}$ are antibodies interstitial fluids (ISF) concentrations in tissues tight ($V_{tight}$ ) and leaky ($V_{leaky}$). The pregnancy related physiological compartments such as placenta and fetus are incorporated in the leaky compartment of the mPBPK model. The The $L$ is total lymph flow equal to the sum of $L_1$ and $L_2$, the lymph flow for $V_{tight}$ and $V_{leaky}$. The σ1 and σ2 are vascular reflection coefficients for $V_{tight}$ and $V_{leaky}$. The σL is the lymphatic capillary reflection coefficient, which is assumed to be 0.2.

**Table S3:** Parameters used for development and evaluation of mPBPK model for Anti-D IgG in pregnant women

| | **Parameters for pregnant women in third trimester** |
|---|---|
| **Plasma volume (L)** | 3.67 |
| **Leaky tissue volume (L)** | 4.37*(BW/70) |
| **Tight tissue volume (L)** | 8.11*(BW/70) |
| **Lymph volume (L)** | 5.2*(BW/70) |
| **Total lymph flow (L/ day)** | 2.9*(BW/70) |
| **Lymphatic capillary reflection coefficient** | 0.2 |
| **Vascular reflection coefficient*** | $\sigma_1$= 0.99<br>$\sigma_2$= 0.81 |
| **Clearance (L/day)** | 0.259*(BW/70)^0.75 |

*$\sigma_1$ & $\sigma_2$ indicate reflection coefficient of tight and leaky tissues, respectively.

The reflection coefficients and clearance are estimated with %CV <10% using PK data of anti-D Ig following intravenous administration in pregnant women (Bichler et al. 2003)[25].

**Figure S1:** Schematic representation of the minimal PBPK (mPBPK) model framework, including model inputs and outputs used for characterizing the pharmacokinetics of IGIV and anti-D Ig in nonpregnant and pregnant women. The mPBPK model categorizes tissues into leaky compartments (liver, kidney, heart, and other highly vascularized organs) and tight compartments (muscle, skin, adipose tissue, and brain) based on vascular permeability characteristics. For pregnancy applications, placenta and fetus were incorporated into the leaky tissue compartment to account for pregnancy-related physiological changes.

## Model Input

- Physiology
- Biochemical
- Dosing

## Model Output

- Conc. vs time
- PK parameters
- Dosing optimization

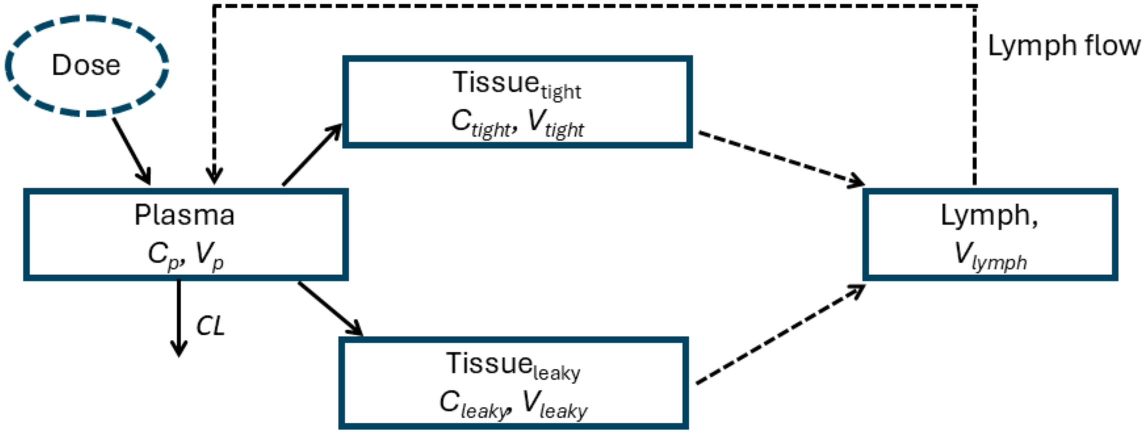

Supplement: Supplementary file 1 [file antibodies-14-00099-s001.zip › antibodies-3934679-supplementary.pdf]
